# Prevalence and factors associated with rotavirus infection among children admitted with acute diarrhea in Uganda

**DOI:** 10.1186/1471-2431-10-69

**Published:** 2010-09-24

**Authors:** Jane S Nakawesi, Eric Wobudeya, Grace Ndeezi, Edison A Mworozi, James K Tumwine

**Affiliations:** 1 Department of Paediatrics and Child Health, School of Medicine, Makerere University College of Health Sciences, P. O Box 7072 Kampala, Uganda; 2 Department of Paediatrics and Child health, Mulago National Referral Hospital, P.O. Box 7051 Kampala, Uganda

## Abstract

**Background:**

Rotavirus remains the commonest cause of severe dehydrating diarrhea among children worldwide. Children in developing countries die more because of several factors including poorer access to hydration therapy and greater prevalence of malnutrition. Hitherto, the magnitude of rotavirus disease in Uganda has remained unknown. This study was therefore done to determine the prevalence and factors associated with rotavirus infection among children aged 3-59 months admitted with acute diarrhea to paediatric emergency ward of Mulago Hospital, Uganda

**Methods:**

Three hundred and ninety children, aged between 3-59 months with acute diarrhoea were recruited. The clinical history, socio-demographic characteristics, physical examination findings and laboratory investigations were recorded. Stool samples were tested for rotavirus antigens using the DAKO IDEIA rotavirus EIA detection kit.

**Results:**

The prevalence of rotavirus infection was 45.4%. On multivariate analysis rotavirus was significantly associated with a higher education (above secondary) level of the mother [OR 1.8; 95% CI 1.1-2.7]; dehydration [OR 1.8; 95% CI 1.1-3.0] and breastfeeding [OR 2.6; 95% CI 1.4-4.0]. Although age was significantly associated with rotavirus on bivariate analysis; this association disappeared on multivariate analysis. No significant association was found between rotavirus infection and nutritional status, HIV status and attendance of day care or school.

**Conclusions:**

Rotavirus infection is highly prevalent among children with acute diarrhoea admitted to Mulago Hospital in Uganda.

## Background

Diarrhea is a leading cause of death in children under five years of age globally, with an estimated 1.5 million child deaths per year[1]. Rotavirus infection remains the commonest cause of severe dehydrating diarrhea among children worldwide [2]. Each year rotavirus causes an estimated 111 million episodes of diarrhea requiring only home care, 2 million hospitalizations and 400,000 deaths in children under 5 years; 82% of which occurs in children in the poorest countries [3,4].

The reported prevalence of rotavirus diarrhea from global surveillance networks and hospital based studies ranges from 6% to 56% [1,3-15].

Despite the similarity in the prevalence of rotavirus diarrhea between the developed and developing countries, there is a variation in the factors associated with rotavirus diarrhea. Factors associated with rotavirus diarrhea in sub-Saharan Africa include; nutritional status, dehydration, episodes occurring in the dry season and age under 2 years [3,13,14]. Some of the studies have found conflicting results on the effect of nutritional status on the risk of rotavirus diarrhea [3,13]. Other studies have found contact with diarrhea outside the household, poor food hygiene, dehydration, education level, accommodation with less than five rooms, bottle-feeding, low birth weight, male gender, maternal smoking, maternal age < 20 years to increase the risk of rotavirus diarrhea [16-19]. Interestingly, so far there has been no association between HIV and rotavirus diarrhea [20].

WHO recommends that rotavirus vaccine for infants be included in all national immunization programmes [21]. Two vaccines, Rotarix™ from GlaxoSmithKline (Rotarix™) and RotaTeq^® ^from Merck & Co have been prequalified by WHO. Rotarix™ was prepared from an individual human strain that replicates well in the intestine. RotaTeq is a combination of five bovine human reassortants that replicates poorly in the gut. Both should be administered orally as two doses for Rotarix™ or three doses for RotaTeq^® ^to babies, with the first dose given at 6-15 weeks of age. Both vaccines are stable at 2-8°C; 2 years for Rotarix™ and 3 years for RotaTeq. Despite the WHO recommendation rotavirus vaccine has not been integrated in the routine immunization programmes in the WHO African region except partial integration in South Africa [22].

This study aimed to determine the prevalence and factors associated with rotavirus diarrhea among children aged under five years in Mulago Hospital, Kampala, Uganda.

## Methods

This cross sectional study was conducted in the paediatric emergency unit of Mulago, Uganda's National Referral and Teaching Hospital; situated in the central region of the country. The unit admits between 30 and 80 patients daily, 10% of whom have acute diarrhea. Mulago hospital also functions as the primary health facility for the surrounding population. The hospital serves mainly the peri-urban and rural populations of Kampala district.

### Clinical methods

Children presenting to the paediatric emergency unit were screened for diarrhea by a nurse. Acute diarrhea is the passage of watery or loose motions 3 or more times in a 24 hours and lasting less than 14 days[23]. Water from a tap, protected well or borehole was considered safe. Proper waste disposal referred to use of a pit latrine or flush toilet. The study was explained to the caregivers and written informed consent obtained. The children were then assessed for eligibility by the study nurse or the principal investigator or the research assistant. Critically ill children with signs of severe dehydration were rehydrated according to the WHO guidelines (100 mls/kg of Ringers lactate infusion; for infants, 30 mls/kg over one hour then 70 mls/kg over five hours and for children 1-5 years, 30 mls/kg over 30 minutes then 70 mls/kg over two and a half hours) before obtaining consent. Children aged 3-59 months with acute diarrhea and where a stool sample could be recovered within 24 hours of hospitalization were immediately recruited into the study after consent by the caregiver. The caregiver was either the biological mother/father or any other legal primary carer for the child. A syringe attached to the rectal tube size 10 was used to aspirate approximately 5 ml of stool from the rectum. Children with bloody diarrhea were excluded.

We measured the weights using a Salter Scale Model 235 6 S for infants and electronic weighing scale model 881 1021659, serial No 1881302074438 S for older children. For easy identification of patients by the research team, each child was assigned a study number marked onto the patient's management chart.

The study was approved by the Makerere University Faculty of Medicine Research and Ethics committee.

### Measurement variables

Data was collected using a pre-coded data collection tool. Variables included child's age, gender, weight, height or length, symptoms, duration of symptoms, feeding practice, water source, home hand washing practices, diarrhea contacts, housing facilities, temperature, hydration status, Rotavirus results and HIV status. We also recorded the caregiver's age, gender, education level and occupation.

### Sampling and laboratory methods

Participants were consecutively enrolled until the required sample size was obtained.

Stool specimens were transported to the laboratory in labeled and securely closed containers. The presence of rotavirus antigens was tested using DAKO IDEIA rotavirus EIA(Enzyme Immunoassay)detection kit (Dako Diagnostics Ltd, Cambridgeshire, UK) within one week of collection. Rotavirus testing was carried out by a laboratory technologist trained in rotavirus detection using standardized operating procedures. The samples were stored in a refrigerator at 2-8°C before testing. Bacteriology was done and the results were given to the doctors immediately for patient management. Of the three hundred and ninety (390) cases, ninety (90) yielded bacteriology results. Of the ninety (90), eighty five (85) yielded Candida albicans, three (3) Escherichia Coli and two (2) Vibrio Cholerae. For the two cases where vibrio Cholerae was isolated the stool sample was negative for the rotavirus antigen. The HIV status of the recruited children was determined using an antibody test (DETERMINE1/2) for children above 18 months and DNA PCR for those below 18 months as part of routine counselling and testing on the ward.

### Statistical analysis

A sample size of 381 children was required for the study using the Kish & Leslie formula [24]for cross-sectional studies, estimating the true prevalence of 54.5%[11]and adopting a precision of 5% with 95% confidence intervals. The data was entered into Epidata 3.1 package and checked for completeness on a daily basis. The clean data was exported to Stata version 10.1 for analysis.

Categorical variables were summarized as frequencies, proportions and continuous variable summarized as means or medians where appropriate with their measures of dispersion.

Bivariate analysis to compare the rotavirus positive and rotavirus negative patient characteristics was done using the Chi-square test or Fisher's exact for categorical variables as appropriate and the student's t test for normal distributed continuous data. Mann-Whitney U test was used to compare skewed continuous data. Child age data was log transformed before further analysis was done.

Factors with a p value ≤ 0.2 at bivariate analysis were entered into a stepwise logistic regression model using the forward hierarchical method to determine those that were independently associated with rotavirus diarrhea. The effect measure used was the odds ratio. *P *values of below 0.05 were considered significant.

## Results

From September 2006 to January 2007, a total of 5230 children were hospitalized in the Mulago hospital paediatric emergency ward, of whom 410 (7.8%) had diarrhea. Of the children with diarrhea, 390 children were recruited into the study. Twenty children were excluded as we could not obtain a stool sample.

Of the eligible children, 355 were less than 24 months. The median age was 10 months (IQR 7 - 16 months) and the mean duration of diarrhea was 4.7 days (SD 2.1). There were more males 235 (60.3%) than females. The median age of the caregivers was 23 years (IQR 20-28). Exclusive breastfeeding was reported in 2.3% of the patients. Ninety three percent reported use of safe water while 89% reported proper waste disposal. The other characteristics are shown in table 1.

**Table 1:** Comparison of Characteristics of study factors by rotavirus status among children admitted with acute diarrhoea.

Factor	Rotavirus positive n [%] [N = 177]	Rotavirus negative n [%] [N = 213]	OR	95% CI	P value
Age of child (Median {IQR})	10 { 7}	11 {11}			0.10*
Age of caregiver (Median {IQR})	24 { 8}	23 { 8}			0.36*
Breast feeding	128 [73.2]	110 [51.6]	2.4	1.5 - 3.7	** < 0.001**
Age < 24 months	I67[94.3]	182 [85.4]	2.8	1.3 - 5.9	**0.004**
Sex (male)	104 [58.8]	131 [61.5]	0.9	0.6 - 1.3	0.58
Caregiver age < 20 years	47 [26.6]	63 [29.6]	0.9	0.6 - 1.3	0.51
Mothers education: Secondary and above	93 [52.5]	81 [38]	1.8	1.2 - 2.7	**0.004**
Occupation: housewife	95[53.6]	119 [55.8]	0.9	0.6 - 1.3	0.66
Household contact with diarrhoea	22 [1.2]	26 [1.2]	1.0	0.6 - 1.9	0.95
Safe water	164 [92.7]	206 [96.7]	0.4	0.1 - 1.0	0.07
Waste disposal: proper	163 [92]	188 [88.2]	1.5	0.7 - 3.0	0.20
4 or more people in house	56 [31.6]	64 [30]	1.0	0.6 - 1.6	0.73
Diarrhoea less than 1 week	175 [98.9]	204 [95.8]	3.8	0.8 - 18.0	0.08
HIV infection	14 [7.9]	22 [10.3]	0.7	0.3 - 1.5	0.40
Dehydration	144 [81.3]	152 [71.3]	1.7	1.08 - 2.8	**0.02**
Wasting (Weight for height z score < -2 SD)	52 [24.4]	41 [23.1]	0.9	0.5 - 1.4	0.70

* Mann Whitney U test

IQR- Interquartile Range

### Prevalence of Rotavirus diarrhea

The prevalence of Rotavirus was 45.4% (95% CI 40.5 - 50.3). The majority of children (95%) with rotavirus diarrhea were less than 24 months.

### Factors associated with Rotavirus diarrhea

At bivariate analysis, breastfeeding, dehydration, age below 2 years and high education level (secondary and above) were significantly associated with rotavirus diarrhea, (table 1). There was no association between HIV and rotavirus diarrhea.

On multivariate analysis, secondary and above education level, breastfeeding and dehydration were independently associated with rotavirus diarrhea (table 2).

**Table 2:** Clinical characteristics independently associated with rotavirus diarrhea among admitted children in Uganda.

Factor	Unadjusted OR(95% CI)	Adjusted OR(95% CI)
**Breast feeding**	2.4 ( 1.5-3.7)	2.6 (1.6 - 4.0)
**Dehydration**	1.7 (1.08 - 2.8)	1.8 (1.1 - 3.0)
**Mothers education: secondary level and above**	1.8 ( 1.2 - 2.7)	1.8 (1.1 - 2.7)

## Discussion

We aimed to determine the prevalence and factors associated with rotavirus diarrhea among hospitalized children aged 3-59 months with acute diarrhea. Hitherto, factors associated with rotavirus diarrhea have not been reported from Uganda.

We found that rotavirus diarrhea accounted for almost half the acute diarrhea hospitalizations in children. The result is however not different from an earlier study in the same hospital in 1987 by Kenya Mugisha [11] implying that the burden of this disease has not changed over the years. Our result is also similar to the sentinel based rotavirus surveillance system and hospital based study results within the African region [1,14]. The hospital-based WHO global networks for surveillance of rotavirus diarrhea report estimated the rotavirus rate to range from 39 - 52% in the African region. A study conducted over a 1-year period in Zambia found a prevalence of rotavirus diarrhea of 24% [13]. Our study largely represents the burden of rotavirus diarrhea in hospitalized children. This hospital-based study result however may not be a true reflection of rotavirus burden in the community or outpatient clinics since the study was conducted during a peak rotavirus season in the study region. Rotavirus positive cases are usually more severe and likely to be more represented in hospital based studies[25].

Factors independently associated with rotavirus diarrhea included: breastfeeding, secondary or higher mother's education level, and dehydration. We found that the breastfeeding children were two and half times more likely to have rotavirus diarrhea after adjusting for age. Breastfeeding has consistently been shown to confer protection against non-viral gastrointestinal pathogens but evidence for viral protection has remained weak [26]. A review of several studies in Bangladesh concluded that breastfeeding is a minor contributor to prevention of rotavirus diarrhea [27]. However, exclusive breastfeeding, particularly in infants, has been shown to offer protection against rotavirus [28]. Due to the small numbers of exclusively breastfed children in our study we could not adequately examine the relationship between exclusive breastfeeding and rotavirus diarrhea. A prospective study in Egypt showed a lower incidence of rotavirus diarrhea in infants fed on breast milk [29] and others have shown evidence that breastfeeding offers protection against severe rotavirus infections only [19,28]. Some prospective studies have found that breastfed infants manifested a milder rotavirus disease [29]. It is possible that breastfeeding may only be protective if it is practiced with an intensity and frequency that allows continuous high level protection of the intestinal mucosa rather than sporadic or low volume feeds [30]. Since most of the children in this study were not exclusively breastfed, they were probably not taking in enough amounts of breast milk. The role of exclusive breastfeeding needs to be explored further in study designed to establish whether exclusive breastfeeding protects against rotavirus diarrhea. In this study children of mothers with a secondary or higher education level were two times more likely to have rotavirus diarrhea. This is in contrast to what was found by Dennehy et al in the USA where children of mothers with a lower education level were more likely to have rotavirus diarrhea [18]. The reasons for this discrepancy are not clear.

Children with dehydration were about two times more likely to have rotavirus diarrhea. Several studies have reported a similar finding [3,29]. This finding is not surprising as rotavirus infection has been associated with severe diarrhea episodes with dehydration elsewhere [3,16].

The high prevalence of rotavirus in the current and other studies in East Africa, reflects the high burden of rotavirus reported from elsewhere in sub Saharan Africa. Although there is a possible seasonal bias in our study conducted during a rotavirus peak season, our results are consistent with the average reported rotavirus detection rates of 45% from the surveillance data on the African region [1]. The exclusion of infants less than three months may have had a limited effect on the prevalence of rotavirus in our study. From the national rotavirus surveillance report, only 2% of recruited children are less than 3 months of age[31]. The rotavirus detection rate in this age group has been reported as low as 2%.

Interpretation of our study results should be with caution given the following limitations; collection of data only over 6 month period hence seasonal problems, no controls against which to gauge the high prevalence and little information on severity of the rotavirus positive cases. Our study may be an overestimate given that it was conducted during the peak season for rotavirus diarrhea Uganda. There was also a possibility of report bias especially on feeding practices, since our study team comprised health workers involved in routine patient care. We tried to minimize this bias by training and sensitization of our research team.

## Conclusion

The burden of rotavirus infection among children less than five years in Mulago hospital in Uganda is high. Factors independently associated with rotavirus diarrhea were high education level (secondary and above) of the mother, breastfeeding, and dehydration.

## Competing interests

The authors declare that they have no competing interests.

## Authors' contributions

JSN conceived the study idea, was the primary study implementer and drafted the manuscript. EW participated in study design, statistical analysis and contributed to the draft manuscript. GN contributed to the draft manuscript. EAM participated in the study design and contributed to the draft manuscript. JKT participated in study design, statistical analysis and contributed to the draft manuscript. All the authors were involved in the interpretation of the results, read and approved the final manuscript.

## Funding

Privately funded by the investigators.

## Pre-publication history

The pre-publication history for this paper can be accessed here:

http://www.biomedcentral.com/1471-2431/10/69/prepub
